# Telomere length reveals cumulative individual and transgenerational inbreeding effects in a passerine bird

**DOI:** 10.1111/mec.13670

**Published:** 2016-05-17

**Authors:** Kat Bebbington, Lewis G. Spurgin, Eleanor A. Fairfield, Hannah L. Dugdale, Jan Komdeur, Terry Burke, David S. Richardson

**Affiliations:** ^1^ School of Biological SciencesUniversity of East AngliaNorwich Research Park, Norwich, Norfolk NR4 7TJUK; ^2^ Department of ZoologyEdward Grey InstituteUniversity of OxfordOxford OX13PSUK; ^3^ School of BiologyThe Faculty of Biological Sciences, University of LeedsLeeds LS2 9JTUK; ^4^ Behavioural Ecology and Physiological GroupGroningen Institute for Evolutionary Life SciencesUniversity of GroningenPO Box 11103, 9700 CC, GroningenThe Netherlands; ^5^ Department of Animal and Plant SciencesUniversity of SheffieldSheffield S10 2TNUK; ^6^ Nature SeychellesPO BOX 1310, MaheRepublic of Seychelles

**Keywords:** heterozygote advantage, inbreeding, lifetime fitness, Seychelles warbler, telomere, trans‐generational effects

## Abstract

Inbreeding results in more homozygous offspring that should suffer reduced fitness, but it can be difficult to quantify these costs for several reasons. First, inbreeding depression may vary with ecological or physiological stress and only be detectable over long time periods. Second, parental homozygosity may indirectly affect offspring fitness, thus confounding analyses that consider offspring homozygosity alone. Finally, measurement of inbreeding coefficients, survival and reproductive success may often be too crude to detect inbreeding costs in wild populations. Telomere length provides a more precise measure of somatic costs, predicts survival in many species and should reflect differences in somatic condition that result from varying ability to cope with environmental stressors. We studied relative telomere length in a wild population of Seychelles warblers (*Acrocephalus sechellensis*) to assess the lifelong relationship between individual homozygosity, which reflects genome‐wide inbreeding in this species, and telomere length. In juveniles, individual homozygosity was negatively associated with telomere length in poor seasons. In adults, individual homozygosity was consistently negatively related to telomere length, suggesting the accumulation of inbreeding depression during life. Maternal homozygosity also negatively predicted offspring telomere length. Our results show that somatic inbreeding costs are environmentally dependent at certain life stages but may accumulate throughout life.

## Introduction

In inbred individuals, increased homozygosity leads to the expression of deleterious recessive alleles and the reduction of any heterozygote advantage, and has been shown to reduce fitness across a broad range of taxa (Keller & Waller 2002; Brekke *et al*. 2010; Simmons 2011; Lacy & Alaks 2013). Inbreeding depression may result through suboptimal cell functioning: both metabolic efficiency (Kristensen *et al*. 2006; Ketola & Kotiaho 2009) and immune responses (Reid *et al*. 2003) decline with increased homozygosity. Disruption to such physiological processes as a result of inbreeding can lead to the increased production, or inefficient processing, of damaging oxidant molecules (Nemoto *et al*. 2000; Balaban *et al*. 2005; Massudi *et al*. 2012), the effects of which are normally mitigated by upregulation of antioxidant production. Inbred individuals may be further limited in their ability to produce antioxidant defences if they are less able to access food and other key energetic resources (Ketola & Kotiaho 2009), for example, through reduced competitive ability (Sharp 1984). We therefore expect inbreeding to reduce fitness. However, the costs of inbreeding reported in natural systems vary hugely among individuals and populations (Armbruster & Reed 2005). This lack of consistency could result from variation in the available power to detect effects (Huisman *et al*. 2016), but may also indicate that certain individuals and populations experience low costs of inbreeding.

There are various reasons why the fitness costs of inbreeding might remain undetected in natural systems (e.g. Keane *et al*. 1996; Kalinowski *et al*. 1999). First, inbreeding depression is usually measured in terms of survival or reproductive success (reviewed in Chapman *et al*. 2000; Walling *et al*. 2011; Kennedy *et al*. 2014). These ultimate components of fitness might, however, be confounded by other factors, such as variation in habitat quality and stochastic mortality (Miller & Coltman 2014). Second, inbreeding depression in offspring could be confounded by parental effects, which may in turn be affected by the inbreeding level of either or both of the parents. Thus, an individual's fitness may be reduced as a result of having inbred parents regardless of its own level of inbreeding (Keller 1998; García‐Navas *et al*. 2014). Such indirect costs of inbreeding could manifest as reduced parental investment by inbred parents, for example through poor prenatal nutrition (Wetzel *et al*. 2012), or the attraction of a poor‐quality mate (Sheridan & Pomiankowski 1997). Third, inbreeding variance in natural populations has often been investigated using individual homozygosity across a panel of neutral molecular markers. The results of such studies are inconsistent (Hansson & Westerberg 2002), which is likely due to the potential inaccuracy of measuring genome‐wide homozygosity using a limited number of markers (Slate *et al*. 2004; Balloux *et al*. 2004; reviewed in Miller & Coltman 2014) – this method is therefore mainly suited to studies of isolated populations with high inbreeding variance (Slate *et al*. 2004). Finally, inbreeding effects are easily confounded by variation in external factors. For example, it may only be possible to detect inbreeding costs during periods of heightened environmental or physiological stress (Keller *et al*. 2002; Marr *et al*. 2006; Auld & Relyea 2009) or when there is sufficient variation in individual success (Harrison *et al*. 2011). Furthermore, any negative effects of stressful periods may be cumulative, so that inbreeding‐related damage accrued during unfavourable conditions builds up in cells and tissues but may only be detectable above a certain threshold level (Grueber *et al*. 2010). Such cumulative effects of inbreeding can only be studied with longitudinal data on environmental conditions, ideally collected across individuals’ entire lifespans. The rarity of such data from wild populations, combined with the potential for somatic damage to remain undetected until survival effects are visible, might create a substantial gap in our understanding of the costs of inbreeding.

The complications in measuring inbreeding depression may be alleviated using telomere dynamics to capture individual variation in inbreeding effects. Telomeres are regions of noncoding DNA that protect chromosomes from DNA damage during meiosis (Blackburn 1991). Telomere loss occurs during cell replication, but is also driven by metabolic oxidant by‐products that damage DNA (Finkel & Holbrook 2000; von Zglinicki 2000). Oxidative stress (an imbalance in favour of oxidant molecules over defensive antioxidant molecules) arises when individuals do not produce sufficient levels of antioxidants (Finkel & Holbrook 2000), often in periods of elevated somatic stress such as during reproduction (Van De Crommenacker *et al*. 2011) or long‐distance travel (Constantini *et al*. 2007). Telomere length, while probably not causative, appears to be linked to cell‐level oxidants and is a useful biomarker for somatic damage (Simons 2015).

Recent studies have linked telomere dynamics to individual life histories and survival in a range of vertebrates (reviewed in Barrett & Richardson 2011), and telomere shortening has been found to reflect energetic costs in relation to factors including reproductive investment (Bauch *et al*. 2013), chronic infection (Asghar *et al*. 2015) and early‐life conditions (Heidinger *et al*. 2012). Given the links between impaired somatic function and inbreeding (e.g. Teska *et al*. 1990; Norman *et al*. 1995) and between rates of telomere shortening and somatic stress (Von Zglinicki 2002; Epel *et al*. 2004), inbred individuals should have shorter telomeres than outbred individuals. Unlike fitness measures such as survival and reproductive success, telomere length reflects exposure to factors that have influenced an individual's intrinsic condition up to any given point in time. For example, if inbreeding depression in the parental generation limits the amount of investment in offspring (reviewed in Keller & Waller 2002), then offspring telomere length should be negatively associated with parental homozygosity, at least in early life when parental investment is key. Similarly, if inbreeding depression mainly manifests during stressful periods (Keller *et al*. 2002; Marr *et al*. 2006; Auld & Relyea 2009), then telomere loss during environmental stress will be greater among inbred than outbred individuals. Furthermore, the difference in telomere loss between inbred and outbred individuals should increase with age as more stressful periods are experienced.

The Seychelles warbler *Acrocephalus sechellensis* provides an excellent system in which to investigate the costs of inbreeding in a natural setting. The population on Cousin Island, Seychelles, has been extensively monitored, with birds regularly caught and sampled, since 1994. Virtually no migration to or from the island occurs (Komdeur *et al*. 2004), creating a small (*ca* 320 adults), closed population with excellent longitudinal data on individual environmental conditions. Inbreeding occurs frequently in the Seychelles warbler, with *ca* 5% of all offspring having parents that are first‐order relatives (Richardson *et al*. 2004). Individual homozygosity, as assessed at a panel of microsatellite loci, does not directly influence adult survival in this species, but in poor environmental conditions, maternal (but not paternal) homozygosity predicts juvenile survival (Richardson *et al*. 2004; Brouwer *et al*. 2007). Importantly, both juvenile and adult telomere length predict survival in the Seychelles warbler (Barrett *et al*. 2013), while juvenile telomere length is also strongly dependent on the year of hatching (L. G. Spurgin, K. Bebbington, E. A. Fairfield, M. Hammers, J. Komdeur, T. Burke, H. L. Dugdale & D. S. Richardson, submitted).

In this study, we investigate how the telomere length of individual Seychelles warblers varies with individual and parental homozygosity to quantify the somatic cost of inbreeding in a natural setting. Specifically, more rapid telomere loss in inbred individuals should lead to a negative relationship between individual homozygosity and telomere length. Parental investment is crucial in altricial bird species such as the Seychelles warbler and, given the extremely long period of offspring dependence in this species (Eikenaar *et al*. 2007) and our previous finding that offspring survival is related to maternal homozygosity (Richardson *et al*. 2004), we also predict that individual telomere length will vary with maternal and paternal homozygosity. Finally, we hypothesize that the relationship between telomere length and homozygosity is environmentally dependent and will accumulate over individuals’ lifetimes.

## Materials and methods

### Study species and system

We use data collected as part of a long‐term study of Seychelles warblers on Cousin Island, Republic of Seychelles (Komdeur 1992; Hammers *et al*. 2013). The Cousin population is saturated at approximately 320 individuals in *ca* 110 territories (Komdeur 1996; Brouwer *et al*. 2009). Each year during the main breeding season (June–September) and in some years during the minor breeding season (January to March), a census is carried out, all breeding attempts are followed, and birth dates are obtained to give accurate age estimates for all individuals in the population. During each season, as many birds as possible are caught using mist nets and (if not already ringed) given a metal BTO ring and a unique combination of three colour rings for individual identification. As a result, many birds are caught on their natal territories as dependent fledglings and subsequently sampled multiple times during their lives. A small (25 μL) blood sample is taken by brachial venipuncture from all captured individuals and stored in 0.8 mL of absolute ethanol. The age class of each bird (fledgling vs. adult) is confirmed using eye colour (Komdeur 1992).

In this study, we used a total of 1064 samples from 592 individuals caught between 1995 and 2009, for which we had both telomere length measures and detailed lifelong ecological data. Our data set included both juveniles (aged under 1 year at sampling: 90 males, 82 females) and adults (aged over 1 year: 248 males, 229 females).

Seychelles warblers defend year‐round territories and their diet consists entirely of insects taken from leaves within the territory (Komdeur 1996). There is annual variation in insect availability on Cousin (Komdeur 1992), which is measured each year as the island‐wide mean number of insects per unit leaf area counted across all territories on the island (termed ‘annual food availability’). As telomere length should be a function of past as well as present experiences, we also calculated mean island‐wide insect food availability (termed ‘lifetime food availability’) across the lifespan of each individual up to the point of sampling.

### Molecular methods

DNA for sexing and microsatellite analysis was extracted from blood samples using ammonium acetate, following Richardson *et al*. (2001). Sex was determined using the PCR method developed by Griffiths *et al*. (1998). To measure individual homozygosity, we used individual genotype data from a panel of 30 polymorphic microsatellite loci previously developed in the Seychelles warbler (Richardson *et al*. 2001; Spurgin *et al*. 2014). Although not all individuals were typed at all 30 loci, 99% were typed at 26 or more loci and 100% were typed at 20 or more loci. To determine parentage, we used the same 30 microsatellites to assign within‐group parentage using maximum‐likelihood estimation in masterbayes 2.52 (Hadfield *et al*. 2006) with Wang's (2004) genotyping error model, following the *MbG_Wang* method of Patrick *et al*. (2012). Genotyping error rates were set to 0.005. We ran 15 001 000 iterations, discarding the first 1000 and applying a thinning interval of 15 000. Autocorrelation between successive iterations was <0.1. Tuning parameters were set to 0.01 for unsampled sires and 0.005 for unsampled dams to ensure the Metropolis–Hasting values ranged from 0.2 to 0.5. To maximize assignment confidence, we used only individuals for which the candidate father (assigned with an acceptance threshold of 80%) was the social partner of the dominant breeding female in the territory. Full details of the parentage assignment protocol can be found in Wright (2014).

We used the r (2014) package rhh 1.0.1 (Alho & Välimäki 2012) to calculate individual standardized heterozygosity between 0 and 2 (Coltman *et al*. 1999; Alho *et al*. 2010). We henceforth refer to homozygosity (i.e. 2 – standardized heterozygosity) in accordance with the hypothesized negative effect of inbreeding on telomere length. Offspring homozygosity at 14 of these markers correlates well with parental relatedness in this species (Richardson *et al*. 2004). We used two methods to test the ability of our extended microsatellite panel (30 loci) to reflect genomewide levels of homozygosity and thus inbreeding. Using the Rhh package in r (Alho & Välimäki 2012), we calculated a mean homozygosity–homozygosity correlation coefficient (genotyped loci are randomly assigned to one of two groups correlated against each other to determine similarity) from 5000 iterations of the correlation (Balloux *et al*. 2004; Alho *et al*. 2010). We also estimated identity disequilibrium (g_2_) from 5000 bootstraps for our typed loci using RMES (David *et al*. 2007).

### Telomere measurement

For telomere measurement, we used quantitative PCR (qPCR), following the reaction protocol developed previously for the Seychelles warbler (Barrett *et al*. 2013). Briefly, DNA was extracted using a DNeasy blood and tissue kit (Qiagen) according to the manufacturer's instructions with modification of overnight lysis at 37 °C and a final DNA elution volume of 80 μL. DNA integrity was verified visually using electrophoresis on a 1.2% agarose gel, and the concentration was quantified using a NanoDrop 8000 Spectrophotometer (ThermoScientific). We used a relative measure of telomere length that describes the amount of telomeric DNA in a sample relative to that of GAPDH, a constantly expressed reference gene. LinRegPCR 2014.2 was used to correct baseline fluorescence, determine the window of linearity for each amplicon and calculate individual well efficiencies. Threshold values (Nq) were set in the centre of the window of linearity per amplicon for all samples. We then calibrated quantification cycle (Cq) values per amplicon across different plates by pooling six blood samples as a ‘golden sample’ interplate calibrator (interplate repeatability for telomere amplicon = 0.94). We calculated the mean Cq value for each sample, excluding samples where Cq values differed by >0.5 between the two repeats. We then calculated relative telomere length (RTL) for each sample using equation 1 in Pfaffl (2001). We chose to use RTL rather than continuing with the previously used method for calculating absolute telomere length (Barrett *et al*. 2013), as (i) using RTL enabled us to run more samples per plate (as an oligo standard is not required) and (ii) most other studies have adopted the RTL method, very few have calculated absolute telomere length (which we developed to allow cross‐species comparisons), and our experience now suggests that such comparisons are unlikely to be reliable.

### Statistical analyses

To investigate the effect of individual and parental homozygosity on RTL, we first constructed minimal models in r 3.2.2 (R Core Team 2014) containing all variables that have been associated with either juvenile or adult telomere length in the Seychelles warbler (detailed below). We then constructed full models by adding homozygosity measures and biologically relevant interactions (separately for individual and parental homozygosities) to the minimal models, but removing any nonsignificant interactions. Effect sizes and *P*‐values (calculated using likelihood ratio tests) for nonsignificant interactions were obtained by re‐introducing them into the final model (reported in Tables 1 and 2). We determined whether the final model better described the data than the minimal model by comparing AICc values, considering differences of >2 to be significant (Symonds & Moussalli 2011). *R* ^2^ values were calculated using mumin (Barton 2013). We checked for collinearity between explanatory variables by calculating variance inflation factors and correlating variables with each other.

**Table 1:** Parameter estimates from models of juvenile relative telomere length in relation to (a) individual (I) homozygosity and (b) maternal (M) and paternal (P) homozygosity

Homozygosity	Model	Parameter	Estimate ± SE	*P*
a) Individual *n *=* *137	Minimal	Annual food availability	0.02 ± <0.01	<0.01
		Sex (male)	0.06 ± 0.05	0.19
	Final	**Homozygosity (I)**	**−1.24 ± 0.47**	**<0.01**
		**Annual food availability**	**−0.07 ± 0.03**	**0.04**
		Annual food availability*Homozygosity (I)	**0.09 ± 0.03**	**<0.01**
		Sex (male)	0.06 ± 0.05	0.23
		Sex*homozygosity (I)	−0.14 ± 0.24	0.57
b) Parental *n *=* *77	Minimal	Annual food availability	0.02 ± 0.01	0.05
		Homozygosity (I)	−0.32 ± 0.17	0.07
		Annual food availability*Homozygosity (I)	0.08 ± 0.05	0.14
	Final	**Annual food availability**	**0.02 ± 0.01**	**0.05**
		Homozygosity (I)	−0.32 ± 0.17	0.07
		Annual food availability*Homozygosity (I)	−0.08 ± 0.05	0.14
		Homozygosity (P)	0.18 ± 0.14	0.20
		Homozygosity (M)	−0.16 ± 0.17	0.35
		Homozygosity (M)*Annual food availability	−0.04 ± 0.05	0.43
		Homozygosity (P)*Annual food availability	<0.01 ± 0.04	0.91

Significant terms in the final models are in bold.

**Table 2:** Parameter estimates from models of adult relative telomere length in relation to a) individual (I) homozygosity and b) maternal (M) and paternal (P) homozygosity

Homozygosity	Model	Parameter	Estimate ± SE	*P* value
a) Individual *n *=* *568	Minimal	Lifetime food availability	0.03 ± <0.01	<0.01
		Age	−0.03 ± <0.01	<0.01
		Sex (male)	0.08 ± 0.03	<0.01
	Final	**Lifetime food availability**	**0.03 ± <0.01**	**<0.01**
		**Age**	**−0.03 ± <0.01**	**<0.01**
		**Sex (male)**	**0.08 ± 0.03**	**<0.01**
		**Homozygosity (I)**	**−0.14 ± 0.07**	**0.04**
		Lifetime food availability*Homozygosity (I)	−0.01 ± 0.02	0.50
		Age*Homozygosity (I)	0.01 ± 0.02	0.61
		Sex*Homozygosity (I)	0.01 ± 0.13	0.93
b) Parental *n *=* *182	Minimal	Lifetime food availability	0.02 ± <0.01	<0.01
		Homozygosity (I)	−0.36 ± 0.12	<0.01
		Age	−0.03 ± 0.01	0.02
		Sex (males)	0.10 ± 0.05	0.05
	Final	**Lifetime food availability**	**0.01 ± <0.01**	**0.01**
		**Homozygosity (I)**	**−0.26 ± 0.12**	**0.03**
		**Homozygosity (M)**	**−0.32 ± 0.10**	**<0.01**
		**Age**	**−0.03 ± 0.01**	**<0.01**
		**Sex (male)**	**0.10 ± 0.05**	**0.03**
		Homozygosity (P)	0.16 ± 0.11	0.17
		Food availability at birth	<0.01 ± 0.01	0.89
		Homozygosity (P)*Food availability at birth	0.05 ± 0.03	0.12
		Homozygosity (M)*Food availability at birth	0.02 ± 0.02	0.51

Significant terms in the final models are in bold.

Telomere length and its predictors are different in adults and juveniles so we tested the relationship between individual homozygosity and RTL separately for juveniles and adults. In all models, RTL, which was normally distributed, was used as a response variable. For juveniles (*n *=* *187), we used general linear models without random effects, because individuals were sampled only once as juveniles. We included annual food availability to incorporate cohort effects in the minimal model. Previous studies in this species have reported sex‐biased inbreeding depression (Richardson *et al*. 2004), so we also included sex in the minimal model to test for an interaction with homozygosity. In the full model, we added individual homozygosity and tested for interactions between individual homozygosity and annual food availability, and homozygosity and sex.

For adults, we used mixed models with ‘individual ID’ as a random effect, because some adults had multiple measurements of RTL (*n *=* *737 samples from 420 individuals). We included age in the minimal model for adults, which is the only variable known to predict adult Seychelles warbler telomere length (Barrett *et al*. 2013). We also included sex, so we could test for sex‐biased effects of homozygosity on RTL in the full model. The effect of lifetime food availability on RTL has not been previously investigated in adult Seychelles warblers but we included it in the minimal model because annual food availability has a strong influence on RTL in early life. In the full model, we added individual homozygosity as a fixed effect and tested for interactions between individual homozygosity and age, individual homozygosity and sex, and individual homozygosity and lifetime food availability.

For a subset of individuals (77 juveniles and 127 adults) for which both parentage and telomere data were available, we tested for an association between maternal and paternal homozygosities and offspring RTL. We first performed a linear model to assess the relationship between offspring and parental homozygosities. We then tested the effects of maternal and paternal homozygosity on RTL separately for offspring sampled as juveniles and adults. Minimal models were constructed as in the previous paragraph but also included any additional predictors arising from the individual homozygosity analyses. In the full models, we added maternal and paternal homozygosity as fixed effects. For juvenile offspring, we also included the interactions between both parental homozygosities and annual food availability. For adult offspring, we included food availability in the first year of life, and interactions with parental homozygosities. This allowed us to determine whether effects of parental homozygosity on adult RTL were driven by early‐life conditions or arose independently in adulthood.

## Results

### Homozygosity–homozygosity correlation and g_2_ estimation

Standardized homozygosity was similar in juveniles and adults (juvenile mean ± SD = 0.97 ± 0.21; adult mean ± SD = 0.99 ± 0.23). The homozygosity–homozygosity correlation was significantly positive (mean Pearson's correlation coefficient ± SE = 0.124 ± 0.001, *P *<* *0.001). The g_2_ parameter estimate was significantly greater than zero (g_2_ ± SD = 0.009 ± 0.003, *P *<* *0.001) – comparable to the mean g_2_ value (0.007 ± 0.022 SD) from a recent meta‐analysis (Miller & Coltman 2014). Together, these results indicate that our panel of microsatellite markers reflects genomewide homozygosity in the Seychelles warbler. These parameters are comparable to that reported in other organisms including trees (Rodríguez‐Quilón *et al*. 2015) and mammals (Annavi *et al*. 2014). A recent review suggested that g_2_ measures should be generally meaningful when g_2_ ≥ 0.005 and *P* ≤ 0.01 (Kardos *et al*. 2014); both criteria are met by our microsatellite panel.

### Individual homozygosity and RTL

**Figure 1:**
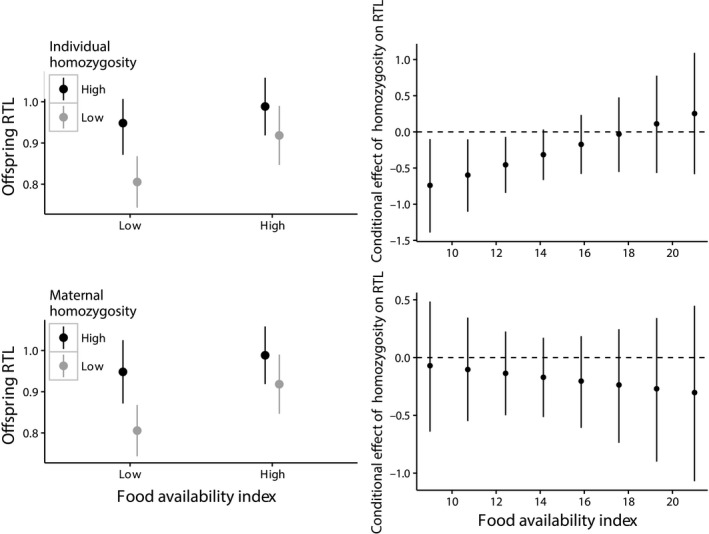
Relationship between standardized individual homozygosity (top row) or standardized maternal homozygosity (bottom row) and relative telomere length of juveniles born in years of high and low food availability, using raw data. In the left‐hand plots, food availability was split into a factor according to the median value for visual clarity, but was modelled as a continuous variable. Right‐hand plots display the conditional effect of homozygosity on RTL, across the range of food availability values. The value on the *y*‐axis indicates the direction of the homozygosity effect on RTL, given the value on the *x*‐axis. Bars represent 95% confidence limits.

In juveniles (*n *=* *137), there was a significant interaction between annual food availability and individual homozygosity (Table 1a): there was a negative effect of individual homozygosity on RTL in years of low food availability but no effect in years of high food availability (Fig. 1a, c). Sex and its interaction with homozygosity were nonsignificant (Table 1a). The final model (*R* ^2^ = 0.11) including individual homozygosity was better supported than the minimal model (Δ AICc = 3.79).

**Figure 2:**
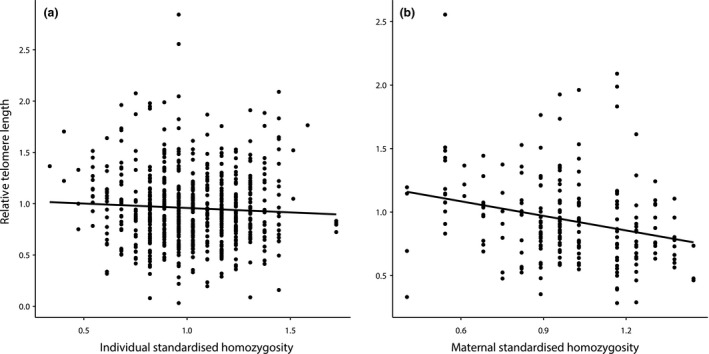
Relationship between (a) standardized individual homozygosity and (b) standardized maternal homozygosity and relative telomere length in adult Seychelles warblers. Points represent raw data, lines represent fitted values (linear regression), and shading represents credible intervals.

In adults (*n *=* *568), RTL was negatively related to individual homozygosity. This relationship between homozygosity and RTL was weak (*R* ^2^ = 0.011; Fig. 2a), but significant (Table 2a). RTL decreased with age, as previously demonstrated in this species, and males had longer telomeres than females (Table 2a). Lifetime food availability was positively related to RTL (Table 2). All interaction terms were nonsignificant and were dropped from the final model. The final model (*R* ^2^ = 0.35) including individual homozygosity did not differ in fit from the minimal model (Δ AICc = 1.22).

### Parental homozygosity and offspring RTL

There was a positive relationship between maternal, but not paternal, homozygosity and offspring homozygosity, but the relationship was weak (maternal: β ± SE = 0.16 ± 0.07, *P *=* *0.03; paternal: β ± SE = −0.01 ± 0.07, *P *=* *0.85). Consequently, both offspring and parental homozygosities could be considered within the same model when testing for relationships between homozygosity and offspring RTL.

In the subset of juveniles with known parentage (*n *=* *77), neither maternal nor paternal homozygosity predicted offspring RTL nor interacted with food availability (Table 1b). There was a nonsignificant trend showing that the RTL of offspring of inbred and outbred mothers differed more in years of low food availability (Fig. 1b), but this was nonsignificant. The null model containing only individual homozygosity (*R* ^2^ = 0.16) was better supported than a model also containing maternal and paternal homozygosity (Δ AICc = 2.37). Finally, in the subset of adults with known parentage (*n *=* *182), maternal homozygosity was negatively related to offspring RTL (Fig. 2b). Neither paternal homozygosity, food availability in birth year nor any interactions significantly predicted offspring RTL. The final model (*R* ^2^ = 0.22) including both individual and maternal homozygosity had a lower AICc than the minimal model (Δ AICc = 3.90).

## Discussion

Our results demonstrate a negative relationship between an individual's homozygosity and relative telomere length, revealing inbreeding costs using a more sensitive measure compared to power‐ and resolution‐limited survival and reproduction measures. In early life, this relationship was dependent on environmental conditions (i.e. annual food availability), whereas inbred adults had shorter telomeres regardless of the food availability they experienced across life. This suggests that the effect of inbreeding on telomeres may accumulate, as more stressful factors are experienced, so that by adulthood shorter RTL is consistently associated with higher homozygosity. Maternal, but not paternal, homozygosity was also linked to adult RTL, indicating trans‐generational impacts of inbreeding. Although the relationships we report are weak, we believe they offer useful insight into the fine‐scale mechanics of inbreeding depression in the wild.

Telomere length is an established biomarker of somatic costs (Hall *et al*. 2004; Ujvari & Madsen 2009; Boonekamp *et al*. 2014) and as such is a good candidate to detect inbreeding depression. Previous studies of inbreeding depression in the Seychelles warbler have reported no relationship between individual homozygosity and survival (Richardson *et al*. 2004; Brouwer *et al*. 2007). This previous underestimation of inbreeding depression in this species probably reflects the fact that inbreeding damage accumulates in cells and tissues; whereas reduced survival may only be detected at some threshold of damage, shorter telomeres can be detected at any point. Thus, our finding that RTL varies with individual homozygosity suggests that inbred individuals have worse somatic condition, which could arise through two nonmutually exclusive pathways. First, inbred individuals may have suboptimal cell functioning that directly increases oxidant levels and increases damage to telomeres (Nemoto *et al*. 2000; Balaban *et al*. 2005; Massudi *et al*. 2012). Second, inbred individuals could mitigate poor cell functioning under normal circumstances, but experience greater‐than‐normal damage during periods of stress due to poor physiological (Armario *et al*. 1995) or behavioural (Bleakley *et al*. 2006) responses.

In line with the second of these pathways, we hypothesized that inbreeding damage to telomeres would be cumulative and vary as a function of the number of stressful events experienced over an individual's entire lifetime. In juvenile Seychelles warblers, the strength of the relationship between inbreeding and telomeres varied with food availability at birth – evidently a key early‐life stressor in this species. By calculating lifetime food availability for adults across their pre‐sampling life and testing for an interaction between this and homozygosity, we hoped to capture some of the variation in stress exposure over life. Adults with lower lifetime food availability have logically experienced more food‐poor periods and the resulting stress accumulation should have impacted inbred birds to a greater extent. Our finding that individual homozygosity and lifetime food availability have consistently negative (rather than interacting) relationships with adult RTL does not support this. This may be because adults face an increased number of different types of stressors, linked to factors such as reproductive effort and social status. An interaction between age and homozygosity on RTL would provide more unequivocal support for the prediction that inbreeding costs accumulate across life, as older adults should have (on average) experienced more (generic) stressors than younger adults. However, there is strong selective mortality of individuals with shorter telomeres in this species (Barrett *et al*. 2013), which likely confounds the interaction between age and inbreeding. Nonetheless, our finding that homozygosity as a main effect is significantly related to telomere length in adults, but not juveniles, provides some evidence that the effect of inbreeding on telomere attrition is cumulative over an individual's lifetime. If early life was the key driver of inbreeding depression, we would expect the interaction between food availability in year of birth and homozygosity to be present even in adulthood. The fact that the negative effect of homozygosity is continuous in adulthood suggests that (multiple) further stressful periods experienced in the post‐juvenile period have compounded the effects of inbreeding that commence in early life.

A previous study on the Seychelles warbler showed that maternal (but not paternal) homozygosity was negatively related to juvenile survival, but that this effect only occurred during low quality breeding seasons and arose through differences in genetics or egg provisioning (Brouwer *et al*. 2007). Although there was no significant interaction between food availability and maternal homozygosity on juvenile RTL, the difference between the RTL of offspring from inbred and outbred mothers when food availability was low (Fig. 1b) was in the same direction as the significant trend with individual homozygosity (Fig. 1a). We may find that with increased sample size and power, the effect becomes significant. We also found that maternal (but not paternal) homozygosity was related to offspring RTL in adulthood. In accordance with individual homozygosity, maternal homozygosity therefore became a consistent predictor of RTL by adulthood. This further supports the idea of accumulating inbreeding costs: as for individual homozygosity, the cost of poor maternal investment [e.g. egg resources which control development (Schwabl 1996)] may reduce an offspring's ability to mitigate costs of external stressors throughout life.

We are only aware of three studies testing the relationship between inbreeding and telomere length. Two studies compared telomere lengths of inbred and outbred strains of laboratory mice and reported extreme elongation of telomeres in inbred strains (Hemann & Greider 2000; Manning *et al*. 2002). These studies considered between‐population rather than within‐population inbreeding variation, and the results cannot easily be compared with those from wild systems. The third study, in a natural population of white‐throated dippers *Cinclus cinclus,* reported no significant relationship between inbreeding and telomere length (Becker *et al*.^2015^), but addressed this only as an aside to questions regarding heritability of telomere length. Given that the study did not consider the potential environmental dependency or cumulative nature of inbreeding effects, it is difficult to make conclusions regarding the reported results. There is a clear need for more tests of individual level inbreeding effects on telomeres in both wild populations and laboratory organisms if we are to understand the impact of inbreeding in the soma.

We show several relationships between RTL and homozygosity at different life stages in this study, but it is important to note that these relationships only explain a limited amount of variation (Figs 1 and 2). They must be confirmed in other systems before any general conclusions about this relationship are drawn. We believe that the low explanatory power of homozygosity arises through the inherent noise in homozygosity measures and also in telomere data. Telomere length is used as a biomarker of biological cost because it is predicted to vary in response to individual physiology, behaviour and environment, but this very useful property means that the relationship with any one given factor is logically weakened by all others. It is extremely difficult to account statistically for all possible drivers of telomere length; laboratory studies where the environmental drivers of telomere length can at least partially be standardized may prove extremely valuable in this sense.

Finally, we present one result that contrasts with previous findings in the Seychelles warbler. We found that adult males had longer telomeres than females, whereas Barrett *et al*. (2013) found no sex difference. The data set used in this manuscript is approximately double the size used by Barrett *et al*. (2013) which, combined with the fact that we report a previously undetected result (rather than failing to support a previously reported result), suggests that our data provide greater power to detect sex differences. Limiting our analysis to only those samples used by Barrett *et al*. (2013) resulted in the relationship between sex and RTL no longer being significant, suggesting that the discrepancy arises through the inclusion of more samples in the current study. Supporting this, the sex effect in our study appears to be more pronounced among cohorts born after 2000 (Fig. S1, Supporting information), which were not included in Barrett *et al*. (2013). It therefore seems likely that the discrepancy between the two studies arises through a combination of difference in power, and potentially some cohort‐level differences.

## Conclusions

To the best of our knowledge, this is the first study to demonstrate a negative relationship between genomewide homozygosity (and thus inbreeding) and telomere length in a natural system. Given the strong link between telomere length and future survival in this and other species, our results suggest that telomeres are able to detect subtle costs of inbreeding that may not be detectable with life‐history data alone. Our results also suggest that inbreeding costs accumulate with age as individuals experience a greater number of stressful periods, but this remains to be tested more thoroughly. Nonetheless, our findings present novel insights into previously unexplored somatic damage that occurs as a result of inbreeding in wild populations.

K.L.B. lead the analysis and drafted the manuscript. E.F. carried out telomere assays and L.G.S. and H.L.D. made substantial contributions to the analysis and interpretation of study data. D.S.R. conceived and directed the study and carried out fieldwork. D.S.R., J.K., H.L.D. and T.B. coordinated the overall Seychelles warbler project. All authors helped draft the manuscript and gave final approval for publication.

## Data accessibility

All morphological data and microsatellite genotypes, along with r scripts used to run analyses, are available in the Dryad Digital Repository, doi: 10.5061/dryad.52fp4.

## Supporting information


**Fig. S1** Sex differences in relative telomere length of Seychelles warblers across years of sampling, showing median (middle line) and second and third quartiles below and above respectively.
