# Supplementary material for: Telomere length reveals cumulative individual and transgenerational inbreeding effects in a passerine bird
Source: Mol Ecol. 2016 May 17;25(12):2949–60. doi: 10.1111/mec.13670 (PMC4999029; doi:10.1111/mec.13670)
Supplement: Supplementary file 1 — Fig. S1 Sex differences in relative telomere length of Seychelles warblers across years of sampling, showing median (middle line) and second and third quartiles below and above respectively. [file MEC-25-2949-s001.docx]

**Telomere length reveals cumulative individual and transgenerational inbreeding effects in a passerine bird**

Kat Bebbington^1^, Lewis G. Spurgin^1,2^, Eleanor A. Fairfield^1^, Hannah L. Dugdale^3,4^, Jan Komdeur^4^, Terry Burke^5^ and David S. Richardson^1,6*^

1. School of Biological Sciences, University of East Anglia, Norwich, UK
2. Department of Zoology, Edward Grey Institute, University of Oxford, Oxford, UK
3. School of Biology, University of Leeds, Leeds, UK
4. Behavioural Ecology and Physiological Group, Groningen Institute for Evolutionary Life Sciences, University of Groningen, Groningen, The Netherlands
5. Department of Animal and Plant Sciences, NERC Biomolecular Analysis Facility, University of Sheffield, Sheffield, UK
6. Nature Seychelles, Mahé, Republic of Seychelles

* Corresponding author: David.Richardson@uea.ac.uk

**Supporting information**

**
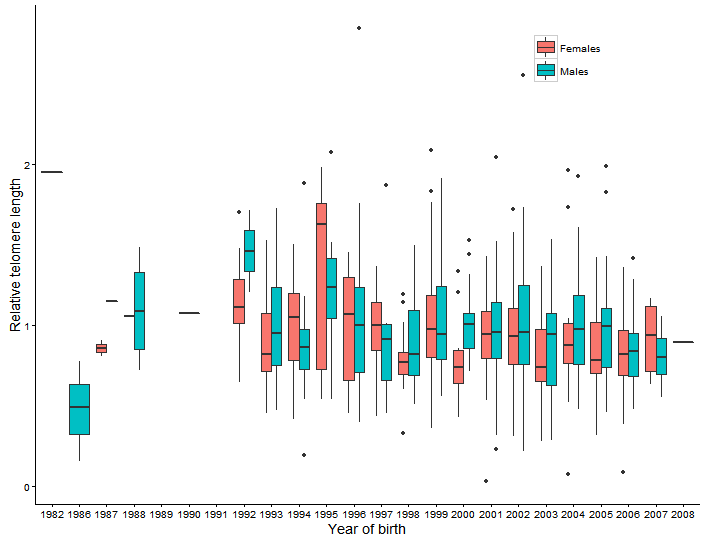
**

**Figure S1** Sex differences in relative telomere length of Seychelles warblers across years of sampling, showing median (middle line) and second and third quartiles below and above respectively.
